# Sense of coherence mediates perceived social support and depressive and anxiety symptoms in cervical cancer patients: a cross-sectional study

**DOI:** 10.1186/s12888-023-04792-y

**Published:** 2023-05-04

**Authors:** Qi Li, Li Liu, Zhihui Gu, Mengyao Li, Chunli Liu, Hui Wu

**Affiliations:** 1 Department of Social Medicine, School of Public Health, China Medical University, No. 77 Puhe Road, Shenyang North New Area, Shenyang, Liaoning Province 110122 China; 2 College of Medical Information, China Medical University, Shenyang, Liaoning Province China

**Keywords:** Cervical cancer, Sense of coherence, Perceived social support, Depressive and anxiety symptoms, Mediating role

## Abstract

**Background:**

Depression and anxiety symptoms are two common psychological disturbances in cervical cancer patients. We tested whether sense of coherence (SOC) mediates the association of perceived social support (PSS) with depression and anxiety symptoms among cervical cancer patients in China.

**Methods:**

We conducted a survey involving 294 cervical cancer patients aged ≥ 18 years from July to December 2020 at three hospitals in Liaoning Province, China; 269 patients completed the survey. We included a demographic questionnaire, the Multidimensional Scale of Perceived Social Support (MSPSS), Antonovsky’s Sense of Coherence Scale, the Hamilton Depression Rating Scale, and the Zung Self-Rating Anxiety Scale (SAS) in this study. We used hierarchical regression analysis to examine the relationship among PSS, SOC, and symptoms of depression and anxiety. We used asymptotic and resampling strategies to explore the mediating effect of SOC.

**Results:**

PSS was negatively associated with depressive symptoms (*r* = − 0.439, *P* < 0.01) and anxiety symptoms (*r* = − 0.325, *P* < 0.01). SOC was negatively related to depressive symptoms (*r* = − 0.627, *P* < 0.01) and anxiety symptoms (*r* = − 0.411, *P* < 0.01). SOC partially mediated the association between PSS and depressive symptoms (a*b = − 0.23, BCa95% CI: [− 0.31, − 0.14]) and anxiety symptoms (a*b = − 0.15, BCa95% CI: [− 0.23, − 0.08]). The proportions of the mediating effect accounting for SOC were 49.78% and 41.73% for depressive symptoms and anxiety symptoms, respectively.

**Conclusion:**

The study showed that SOC could mediate the association between PSS and symptoms of depression and anxiety. This suggests that SOC might serve as a potential target for intervention in symptoms of depression and anxiety that accompany cervical cancer.

## Introduction

Cervical cancer is the most common female gynecologic cancer. In 2020, 604,127 newly diagnosed cases and 341,831 deaths were reported globally [1]. In China, the incidence and mortality of cervical cancer are gradually increasing, and cervical cancer tends to occur in younger women [2]. Cervical cancer is the fourth most common cancer among women worldwide, with an estimated 14,000 new cases and 4,000 cervical cancer-related deaths in the United States in 2022 [3]. Resection is a predominant and effective method of treatment for cervical cancer, after which adjuvant chemotherapy or radiotherapy is sometimes required according to the patient’s tumor burden [4–6]. However, these treatments are often accompanied by unavoidable physical problems such as poor sexual function and surgical trauma, as well as great psychological worries such as self-blame and fear of disease recurrence, which could lead to distress, discomfort, and anxiety in and worse postoperative experiences for cervical cancer patients [7, 8]. Therefore, a large number of cervical cancer patients suffer from anxiety and depression, leading to a decline in their quality of life [8]. Therefore, it is necessary to pay attention to improving the mental health of patients after cervical cancer surgery.

Some studies have shown that depression and anxiety are two common psychological disorders in cancer patients [9]. Our previous meta-analysis showed that the prevalence of depression (54.90% vs. 17.50%) and anxiety (49.69% vs. 18.37%) was significantly higher in Chinese cancer patients than in noncancer patients [10]. Among gynecological cancer patients, cervical cancer patients had the worst emotional distress and quality of life scores [11–14]. A longitudinal study showed that the prevalence of depression and anxiety in cervical cancer patients at baseline ranged from 7.4 to 11.4% and 53.4–62.9%, respectively [15]. Many studies have focused on variables that influence depression and anxiety in cancer patients because of the high prevalence and negative effects of depression and anxiety symptoms. In addition to the influence of demographic and clinical variables on depression and anxiety, positive psychological factors have begun to receive increasing attention in cancer research in the past 20 years [15, 16]. Based on the literature review, we found that variables such as perceived social support (PSS) and sense of coherence (SOC) were key research topics in this field [22, 28].

In general, “social support” refers to help and protection given to others, especially on an individual basis. One of the most effective means of coping with challenging life events is PSS, which determines a person’s health and well-being [17, 18].

The main effect model theory of social support [19] indicates that social support has a general beneficial effect. Regardless of whether an individual is facing a stressful situation, a good social support system has a positive effect on their mental health. In the main effect model, social support acts on individual mental health in two ways. One way is by providing sufficient material, sufficient information, scientific lifestyle information, correct behavior information, etc., as external resources to directly maintain individual physical and mental health. Some studies have shown that social support has negative correlations with depressive and anxiety symptoms [20–22]. Having a good social support system can prevent depressive and anxiety symptoms. Fisher et al. [23, 24] concluded that social support was strongly associated with depressive and anxiety symptoms in patients with breast cancer and ovarian cancer. They indicated that to help patients reduce the risk of developing depressive and anxiety symptoms, optimizing the social support level might be a good intervention method. In turn, depressive and anxiety symptoms may affect social support. A study suggested that the relationship between PSS and depression was bidirectional [25]. Moreover, some studies have indicated that social support correlates with recovery from depressive and anxiety symptoms [26–28].

The other way that health is influenced in the main effect model of social support is by meeting an individual’s psychological needs, preserving enough positive psychological capital, and maintaining mental health indirectly through improving internal resources [31, 32]. SOC is a core concept of the salutogenic model of health. It reflects an individual’s general perception and his or her internal feelings, which involves inner stability and confidence [34]. We propose that sense of coherence is a mediator of the effects of social support on depressive and anxiety symptoms based on the concept of positive psychology [29, 30, 33], which refers to an individual’s ability to adapt positively to the environment when coping with negative events such as trauma and to mobilize psychological resources to face stress with a positive coping attitude, thus reducing negative effects. Many factors influence SOC, including social support. Previous studies have revealed that perceived social support is positively associated with the sense of coherence [35–38]. Some longitudinal studies have supported this finding [39–41]. In a one-year prospective study, Skärsäter et al. found that PSS is an important cornerstone in the restoration of a person’s SOC. It can be used in interventions that include a patient’s family or close social network in combination with support to assist the patient in improving their SOC [39]. A longitudinal study of people with mental health problems indicated that improving social support with a special emphasis on opportunities for nurturance might provide important contributions for increasing the sense of coherence [40]. Moreover, a five-year follow-up study indicated that PSS appears to be an important component of SOC changes among residents of nursing homes (NH) [41]. Some studies showed that offering greater support to people may substantially strengthen their sense of coherence [35, 38].

It has also been well documented that sense of coherence is often negatively correlated with depressive and anxiety symptoms [42, 43]. Even minor levels of depression are related to weaker SOC [41, 44]. Higher SOC is associated with higher mental health, higher affective well-being, and lower depression levels. In the past few years, with the development of positive psychology, many academics have begun to pay attention to positive psychological changes in individuals after experiencing traumatic events [33]. SOC could act as a positive coping resource for cancer patients with depression [45]. People with a higher level of SOC have reported fewer mental symptoms, such as anxiety and depression, and the prevalence of depression in this group is significantly reduced [46]. Because social support can improve SOC and SOC could act as a positive coping resource for cancer patients with depression [45], SOC may play a mediating role in the relationship between social support and depressive and anxiety symptoms. SOC has also been proposed as a mediator in the relationship between perceived stress and depression in breast cancer patients [47], and Pasricha et al. [48] found that the relationship between perceived social support and mental health was mediated by sense of coherence, which further suggested that SOC may play a mediating role in gynecological cancer patients.

**Fig. 1:**
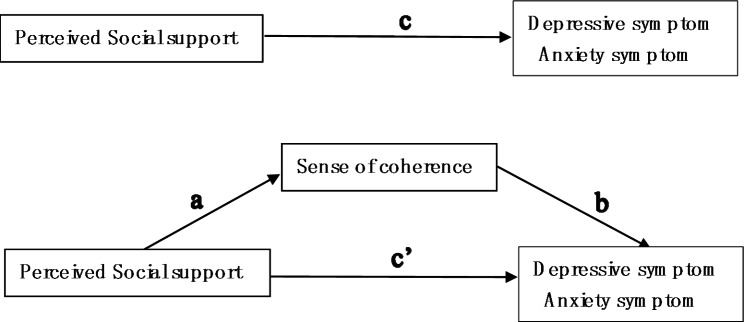
Theoretical model of the mediating role of sense of coherence (SOC) in the relationship between perceived social support (PSS) and depressive and anxiety symptoms. (a) The associations of PSS and SOC; (b) the correlation of SOC with depressive and anxiety symptoms after controlling for the predictor variable; (c) the correlation of PSS with depressive and anxiety symptoms; (c’) the correlation between PSS and depressive and anxiety symptoms after adding SOC

Judging from the literature, the current domestic research tends to analyze the relationship among PSS, SOC, and symptoms of depression and anxiety in a single visual way. Few studies have comprehensively explored the relationship among these three variables in cervical cancer patients. On the other hand, previous studies were limited to negative outcomes such as symptoms of depression and anxiety. They ignored the large and sustainable impact of positive psychological resources on negative outcomes. Under this background, we hypothesized that SOC would mediate the relationship between PSS and symptoms of depression and anxiety in cervical cancer patients, as shown in Fig. 1.

## Materials and methods

### Ethical statement

The study protocol was in accordance with ethical standards and approved by the Ethics Committee of China Medical University. The participants took part voluntarily, and their identities remained anonymous. We protected their privacy and maintained the confidentiality of personal records when processing personal data.

### Study design and sample

We performed a cross-sectional survey in Liaoning Province, China, from July to December 2020. We recruited patients with cervical cancer from 3 hospitals, which were important providers of cancer treatment services in Liaoning Province. A random sampling method was adopted in this study. The subjects of this study were continuously selected from those who met the inclusion criteria. The eligibility criteria were (1) at least 18 years old; (2) pathologically proven cervical cancer; and (3) provided informed consent and voluntary participated. The exclusion criteria were (1) other serious physical diseases and (2) recent major traumatic events. All eligible patients were invited to participate by their oncologists or physicians. The survey instrument consisted of four questionnaires, and a total of 294 patients were enrolled. Ultimately, we received complete responses from 269 cervical cancer patients, with an effective response rate of 91.5%.

### Demographic characteristics

We examined age, education level, marital status, and family monthly income (RMB: Yuan) in this study. We divided age into three ranges: ≤ 45, 46–55, and > 55 years. Options for marital status included “married/living with a partner” and “single/widowed/divorced.” We categorized education levels as “middle school or below” and “junior school and above”. We divided income into two levels: <3000 and ≥ 3000 RMB.

### The measurement of symptoms of depression and anxiety

We chose the Chinese version of the Center for Epidemiologic Studies Depression Scale (CES-D) to measure depression symptoms [49]. Participants were asked to rate the number of times in the previous week that they had experienced depressive symptoms (e.g., low mood, anhedonia, lack of appetite, difficulty concentrating). This questionnaire included 20 items. Each item had 4 responses, ranging from 0 (never) to 3 (always), and overall scores ranged from 0 to 60. The CES-D was used to survey symptoms over the span of one week. A higher average score represented a higher level of depression. A standard CES-D score ≥ 16 indicated depressive symptoms. The Cronbach’s alpha coefficient for the CES-D was 0.905 in this study.

We evaluated anxiety symptoms using the Chinese version of the Zung Self-Rating Anxiety Scale (SAS) [50]. This scale contained 20 items, with each item scored from 1 (never) to 4 (always). The standard total score was obtained from the raw total score via the following formula: standard total score = int (1.25*raw total score). Higher scores represented higher anxiety symptoms. The scale has good reliability and validity and has been widely used in Chinese populations [51]. In this study, the Cronbach’s alpha coefficient for the Zung SAS was 0.850.

### Measurement of perceived social support

We chose the Multidimensional Scale of Perceived Social Support (MSPSS) [52] to assess the PSS of cervical patients. The Chinese version of this scale has good reliability and validity [71]. The MSPSS contained 12 items. The score of each item ranged from 1 (strongly disagree) to 7 (strongly agree). The higher the total score, the higher the level of PSS. In our study, the Cronbach’s alpha coefficient was 0.910.

### The measurement of sense of coherence

The Sense of Coherence Scale was developed by Antonovsky [54]. It consisted of three dimensions (comprehensibility, manageability, and meaningfulness) and had 13 items. This scale was used to assess the internal stability of individuals. The items were measured on a 7-point Likert scale ranging 1 to 7. The scale had a total score of 13 to 91. The higher the total score, the stronger the SOC [55]. In our study, the Cronbach’s alpha coefficient of SOC was 0.807.

### Clinical condition variables

We assessed three clinical condition factors, including new diagnosis, the presence of metastasis, and cancer stage. We divided new diagnosis and the presence of metastasis into responses of “yes” and “no.” We divided cancer stage into three ranges: “I,” “II,” and “III + IV.”

### Statistical analysis

We processed all analyses using IBM SPSS Statistics 21.0 (IBM, Asia Analytics Shanghai). We regarded statistical significance as a two-tailed *p* value < 0.05. We described demographic and clinical characteristics using the mean, standard deviation (SD), number (*n)*, and percentage (%). We compared the differences in depressive and anxiety symptoms among each demographic and clinical group via t tests and one-way analysis of variance (ANOVA). We employed Pearson correlation to examine correlations among the continuous variables. We used hierarchical regression analysis to explore the relationship of PSS and SOC with depressive and anxiety symptoms and explore the possibility of a mediating role of SOC in the relationship between PSS and depressive and anxiety symptoms. We utilized the PROCESS macro (version 3.0 by Andrew F. Hayes) for SPSS to calculate the size of the mediating role and test the hypothesis. To verify whether the mediating effect of SOC was statistically significant with 5,000 bootstrap samples [53]. The differences in scale scores were explained by standardized total scores. We considered significant variables to be control variables. The independent variable was PSS, with depressive and anxiety symptoms serving as the outcomes and SOC as the mediator variable. The “c path” refers to the relationship between PSS and symptoms of depression and anxiety, while the “a*b path” represents the mediating role of SOC. If the absolute value of the “c’ path” coefficient shrinks more than that of the “c path,” a mediating role of SOC may exist. It is only when the confidence interval of the indirect effect does not contain zero that a mediating effect is thought to exist.

## Results

### Descriptive statistics

Table 1 presents the demographic and clinical characteristics of the participants. Among the 269 respondents whose ages ranged from 27 to 77 years, the average age was 53.45 ± 9.35 years. Most of the patients (94.3%) were married or living with a partner, and 68.0% had received a middle school education. In relation to the clinical variables, a minority of the participants (23.1%) were diagnosed at cancer stages III and IV, and 88.1% were newly diagnosed patients. Approximately 70.6% of the participants were free of metastases.

As shown in Table 1, the effect of marital status on depressive symptoms was statistically significant (*p <* 0.05). There was no significant difference in the effect of age, education level, income level, the presence of metastasis, new diagnosis, or stage of cancer on depressive symptoms in the descriptive statistics (*p* > 0.05). The descriptive statistics showed that the effects of age on anxiety symptoms were statistically significant (*p* < 0.05). Education level, marital status, income level, the presence of metastasis, new diagnosis, and cancer stage made no significant difference in the influence of anxiety symptoms (*p* < 0.05).

**Table 1:** Demographic and clinical characteristics (N = 269)

Demographic variables	*n* (%)	Depressive symptoms			Anxiety symptoms		
		Mean ± SD	*F/t*	*P*	Mean ± SD	*F/t*	*P*
**Age (Years)**			2.753	0.066		5.658	0.004
≤ 45	52(19.3)	28.77 ± 11.91			56.48 ± 13.97		
46–55	91(33.8)	31.35 ± 9.78			61.62 ± 11.00		
> 55	126(46.9)	28.07 ± 10.02			56.81 ± 10.30		
**Education level**			1.337	0.182		0.794	0.428
Middle school or below	183(68.0)	29.89 ± 9.12			58.75 ± 10.24		
Junior school and above	86(32.0)	28.08 ± 12.67			57.56 ± 13.89		
**Marital status**			4.882	0.028		3.806	0.052
Married/living with a partner	253(94.3)	29.60 ± 10.12			58.73 ± 11.26		
Single/widowed/divorced	16(5.7)	24.81 ± 13.73			52.75 ± 14.25		
**Income (Yuan per month)**			-0.370	0.712		-1.560	0.120
< 3000	172(64.0)	29.14 ± 9.78			57.55 ± 11.35		
≥ 3000	97(36.0)	29.63 ± 11.46			59.82 ± 11.73		
**New diagnosis**			1.035	0.310		0.004	0.951
Yes	237(88.1)	28.48 ± 10.43			57.54 ± 11.41		
No	32(11.9)	35.53 ± 7.86			64.56 ± 10.57		
**Metastasis**			1.735	0.189		0.379	0.539
No	190(70.6)	29.59 ± 10.24			59.14 ± 11.20		
Yes	79(29.4)	28.66 ± 10.82			56.53 ± 12.12		
**Cancer stage**			1.978	0.140		1.732	0.179
I	60(22.3)	28.27 ± 13.87			57.43 ± 14.57		
II	147(54.6)	28.79 ± 8.49			57.76 ± 9.87		
III + IV	62(23.1)	31.58 ± 10.46			60.74 ± 11.71		

### Correlations among the continuous variables

We computed Pearson’s correlation coefficients among PSS, SOC, depressive symptoms, and anxiety symptoms. As shown in Table 2, depressive and anxiety symptoms were negatively associated with PSS and SOC.

**Table 2:** Mean, SD, and correlations among the continuous variables

	Mean ± *SD*	1	2	3	4	5
1 Age	53.45 ± 9.35	1				
2 PSS	59.43 ± 9.87	-0.029	1			
3 SOC	55.07 ± 10.07	0.064	0.424**	1		
4 Depressive symptoms	29.32 ± 10.40	-0.116	-0.439**	-0.622**	1	
5 Anxiety symptoms	58.38 ± 11.52	-0.077	-0.324**	-0.409**	0.837**	1

**Note**: PSS: perceived social support. SOC: sense of coherence

**p < 0.01 (two-tailed)

### Hierarchical regression analysis

Table 3 shows the results of the association between social support and depressive symptoms by hierarchical regression analysis. First, according to the results of univariate analysis, we added age and marital status (*P* < 0.1) as control variables. In the second step, PSS was added. Finally, we added SOC. After adjusting for age in Step 2, PSS could negatively predict depressive symptoms (*β*= −0.435, *P* < 0.01). PSS accounted for an additional 18.7% of the variance in the dependent variable. In Step 3, SOC negatively predicted depressive symptoms (*β*= −0.520, *P* < 0.01), which explained an additional 21.7% of the variance. After adding SOC, the absolute value of the regression coefficient of PSS on depressive symptoms decreased from 0.435 to 0.218. Thus, SOC might play a mediating role in the relationship between PSS and depressive symptoms in cervical cancer patients.

**Table 3:** Hierarchical linear regression for exploring the variables associated with depressive symptoms

	*F*	*AdjustR²*	Δ *R²*	*B* (95% CI)	SE	*β*	*t*	*P*
**Step 1**	3.994	0.022	0.029					
Age				-0.147(-0.281, -0.014)	0.068	-0.132	-2.174	0.031
Marital status				-5.534(-10.799, -0.270)	2.674	-0.126	-2.070	0.039
**Step 2**	24.41	0.028	0.187					
Age				-0.155(-0.275, -0.035)	0.061	-0.140	-2.546	0.011
Marital status				-3.681(-8.441,1.079)	2.418	-0.084	-1.523	0.129
PSS				-0.458(-0.572, -0.345)	0.058	-0.435	-7.960	0.000
**Step 3**	50.574	0.425	0.217					
Age				-0.104(-0.207, -0.001)	0.052	-0.094	-1.997	0.047
Marital status				-1.480(-5.557,2.598)	2.071	-0.034	-0.715	0.476
PSS				-0.230(-0.336, -0.123)	0.054	-0.218	-4.254	0.000
SOC				-0.537(-0.642, -0.432)	0.053	-0.520	-10.067	0.000

**Note**: PSS: perceived social support. SOC: sense of coherence

Marital status: Married/living with a partner vs. Single/widowed/divorced

Table 4 shows the results of the association between social support and anxiety symptoms by hierarchical regression analysis. After adjusting for age and marital status (*P* < 0.1) in Step 2, PSS negatively predicted anxiety symptoms (*β*= −0.326, *P* < 0.01). PSS accounted for an additional 10.6% of the variance of the dependent variable. In Step 3, SOC negatively predicted anxiety symptoms (*β*= −0.325, *P* < 0.01), which explained an additional 8.6% of the variance. When we added SOC, the absolute value of the regression coefficient of PSS on anxiety symptoms decreased from 0.326 to 0.188. Thus, SOC could probably function as a mediator in the association of PSS with anxiety symptoms in cervical cancer patients.

**Table 4:** Hierarchical linear regression for exploring the variables associated with anxiety symptoms

	*F*	*AdjustR²*	Δ *R²*	B (95% CI)	SE	*β*	*t*	*p*
**Step 1**	1.613	0.002	0.006					
Age				-0.117(-0.265, 0.031)	0.075	-0.095	-1.553	0.122
Marital status				-6.659(-12.415, -0.723)	2.969	-0.135	-2.213	0.028
**Step 2**	16.838	0.106	0.106					
Age				-0.123(-0.264,0.018)	0.071	-0.100	-1.724	0.086
Marital status				-5.077(-10.654, 0.501)	2.833	-0.104	-1.792	0.074
PSS				-0.369(-0.502, -0.236)	0.067	-0.316	-5.470	0.000
**Step 3**	21.865	0.189	0.086					
Age				-0.089(-0.224, 0.046)	0.069	-0.072	-1.295	0.196
Marital status				-3.591(-8.946,1.764)	2.720	-0.074	-1.320	0.188
PSS				-0.215(-0.355, -0.075)	0.071	-0.184	-3.028	0.003
SOC				-0.362(-0.500, -0.224)	0.070	-0.317	-5.171	0.000

**Note**: PSS: perceived social support. SOC: sense of coherence

Marital status: Married/living with a partner vs. Single/widowed/divorced

### Mediating role of SOC

Table 5 presents the path coefficients a (between PSS and the mediator) and b (between the mediator and depressive symptoms), as well as the a*b products. The effect of PSS on the SOC was 0.425. In line with the results from hierarchical multiple regression analysis, SOC was significantly and negatively associated with depressive symptoms after controlling for age, marital status, and PSS. Each BCa 95% CI for a*b of SOC, excluding 0, indicated that it had a significant mediating effect when it was added to the model. Hence, we found a significant mediating role of SOC in the association between PSS and depressive symptoms in cervical cancer patients. Formula (a*b/c) was used to calculate the proportion of the mediating effect. The mediating effect of PSS on physical depressive symptoms was 49.78%.

**Table 5:** PROCESS macro analysis of the mediating effect of SOC on the relationship between PSS and depressive symptoms

	*β*	SE	*t*	*p*	95% CI
Total effect (c)	−0.458	0.057	−7.961	< 0.001	(-0.571, -0.345)
Direct effect (c’)	−0.229	0.054	−4.25	< 0.001	(-0.336, -0.123)
a	0.425	0.056	7.534	< 0.001	(0.314,0.537)
b	-0.536	0.053	−10.066	< 0.001	(-0.641, -0.431)
Indirect effect (ab = c –c’)	− 0.228	0.043		< 0.001	(-0.313, -0.141)

**Note**: BCa 95% CI: the bias-corrected and accelerated 95% confidence intervals; age and marital status were covariates

The path coefficients a (between PSS and the mediator) and b (between the mediator and anxiety symptoms), as well as the a*b products, are presented in Table 6. The effect of PSS on SOC was 0.426. Consistent with the results from hierarchical multiple regression analysis, SOC was significantly and negatively associated with anxiety symptoms after controlling for age, marital status, and PSS. Each BCa 95% CI for a*b of SOC, excluding 0, indicated a significant mediating effect when it was added to the model. Therefore, the significant mediating role of SOC in the association between PSS and anxiety symptoms was revealed among patients with cervical cancer. We used the formula (a*b/c) to calculate the proportion of mediation roles. The mediating effect of PSS on physical anxiety symptoms was 41.73%.

**Table 6:** PROCESS macro analysis of the mediating effect of SOC on the relationship between PSS and anxiety symptoms

	*β*	SE	*t*	*p*	95% CI
Total effect (c)	−0.369	0.067	−5.470	< 0.001	(-0.502, -0.236)
Direct effect (c’)	-0.215	0.071	−3.028	< 0.001	(-0.355, -0.075)
a	0.426	0.057	7.535	< 0.001	(0.315,0.537)
b	-0.362	0.070	−5.172	< 0.001	(-0.500, -0.224)
Indirect effect (ab = c –c’)	0.154	0.038		< 0.001	(-0.228, -0.075)

Note: BC 95% CI the bias-corrected and accelerated 95% confidence interval; Age and income level were covariates

## Discussion

In our study, we found no significant difference in the clinical variables regarding depressive and anxiety symptoms. However, some studies found that cancer stage is a disease-related factor influencing depression and anxiety [56–58]. Hanprasertpong et al. [59] revealed that there is no significance in cancer stage and diagnosis. Yang et al. [57] found that metastasis can’t predict depression and anxiety. Possible reasons for these inconsistent findings could be differences in patients’ characteristics and measurement tools.

We found that PSS was a positive coping resource for symptoms of depression and anxiety among cervical cancer patients, which was consistent with previous findings among various populations [47, 60–63]. Cervical cancer patients may worry about the impact on their personal health and daily life and may have negative emotions [7, 8]. Social support can promote positive behavior and reduce negative emotions [61]. The main effect model of social support plays a general role in the maintenance of individual mental health [19]. The higher the level of social support, the more an individual can accept the changes in disease and the less psychological pressure [47, 62, 63]. Family members and friends, as important supporters of patients’ emotions, can help reduce the pressure of the disease by increasing life help and care, thus preventing the occurrence of depressive and anxiety symptoms in patients. Therefore, a high level of social support can alleviate the negative impact of the pressure of disease, thereby reducing the generation of depressive and anxiety symptoms in patients.

Both previous studies and our findings strongly suggest that social support is significantly associated with a higher SOC [34–37, 64]. Similar to other studies [35, 38, 65], we found that social support may be an important cornerstone in the restoration of a person’s sense of coherence. Social support provides resources for people to cope with various challenges in life, and it can satisfy the psychological needs of an individual, affect internal stability, and improve internal resources. SOC is an important psychological resource that can be strengthened by social support [35, 38].

In addition, our results showed that SOC was negatively correlated with depressive and anxiety symptoms, which is in line with prior outcomes [66, 69]. The same conclusion was made for patients with breast and lung cancer, despite different samples [67, 68]. Schmuck et al. found that strong SOC emerged as the strongest predictor for less severe symptoms of anxiety and depression among HCWs [43]. A previous study investigated 170 spousal caregivers, and high SOC was found to be a protective factor against depression [66]. Individuals with high SOC are better at coping with various pressures (such as diseases, work pressures or adverse social environments) in an effective and flexible way, reducing the damage of stress to health by reducing the adverse physiological reactions and negative emotions related to stress perception.

We found that SOC mediated the relationship between social support and depressive and anxiety symptoms, indicating that low levels of social support are likely to lessen cervical cancer patients’ anxiety and depressive symptoms via their SOC levels. In the main effect model of social support, social support indirectly improves mental health by improving internal mental resources, including SOC. According to Kase et al. [31], SOC mediates the relationship between social support and depressive and anxiety symptoms in Japanese university students, which further supports this mechanism and suggests that SOC may mediate the relationship between social support and depressive and anxiety symptoms in cervical cancer patients. According to previous studies [37, 70], SOC can also act as a mediator among other variables, such between perceived stress and depression among older stroke patients [70] and between social support and self-management among hemodialysis patients [37]. These results further prove the mediating role of SOC. For this reason, medical staff should actively evaluate the level of SOC in newly diagnosed cervical cancer patients to prepare for early intervention.

Based on the above research, we propose some suggestions for clinical work. Many cancer patients have difficulty easily and freely maintaining a normal life under the influence of low PSS and are eager to obtain help. Thus, in caring for patients with cervical cancer, we should pay attention to their existing social support system, guiding patients to actively seek potential social resources and strengthening communication among patients and family members, friends, colleagues, and health care workers. Moreover, medical staff should pay attention to guiding patients to correctly understand their disease and relieve their anxiety and depression.

Our study has several limitations. First, this was a cross-sectional study. Therefore, we were unable to confirm the exact causal relationship among the study variables. Second, we recruited only 269 cervical cancer patients from hospitals in Liaoning Province, China. Third, we did not examine all the relevant factors. Despite the limitations, we have obtained important evidence on the effects of PSS on depressive and anxiety symptoms in Chinese cervical cancer patients. We also tested whether SOC mediates the effect of PSS on symptoms of depression and anxiety using the bootstrapping method. In the future, we will conduct further research recruiting cervical cancer patients from the western and southern parts of China to include a more diverse sample. We will also use longitudinal design methods to infer causality, and we will study more related factors.

## Conclusions

In sum, in cervical cancer patients, PSS and SOC were negatively correlated with depressive and anxiety symptoms. SOC played a mediating role in the relationship between PSS and depressive and anxiety symptoms. Strategies and measures to improve SOC may protect against the impact of PSS on depressive and anxiety symptoms in cervical cancer patients.

## Data Availability

The datasets generated and/or analyzed during the current study are not publicly available due to privacy restrictions but are available from the corresponding author upon reasonable request.
